# Nisin Variants Generated by Protein Engineering and Their Properties

**DOI:** 10.3390/bioengineering9060251

**Published:** 2022-06-10

**Authors:** Yue Zheng, Yuhui Du, Zekai Qiu, Ziming Liu, Jianjun Qiao, Yanni Li, Qinggele Caiyin

**Affiliations:** 1 School of Chemical Engineering and Technology, Tianjin University, Tianjin 300072, China; zhengyuer@tju.edu.cn (Y.Z.); qzk13459107778@163.com (Z.Q.); liuziming@tju.edu.cn (Z.L.); jianjunq@tju.edu.cn (J.Q.); liyanni@tju.edu.cn (Y.L.); 2 Collaborative Innovation Center of Chemical Science and Engineering, Tianjin 300072, China; 3 Key Laboratory of Systems Bioengineering, Ministry of Education, Tianjin 300072, China; 4 MOE International Joint Research Laboratory on Synthetic Biology and Medicines, School of Biology and Biological Engineering, South China University of Technology, Guangzhou 510006, China; duyuhui_107@163.com

**Keywords:** nisin, antibiotics, bioengineering, dairy preservation

## Abstract

Nisin, a typical lantibiotic, has robust antimicrobial activity combined with limited cytotoxicity, and the development of resistance to it is slow. These properties make nisin a promising antimicrobial agent to control pathogenic microorganisms in dairy foods. However, its low solubility, poor stability and short half-life at neutral pH limit its application within the dairy industry. Protein engineering technology has revealed the potential of modifying nisin to improve its properties, and many valuable variants have emerged. This review summarizes progress in the generation of nisin variants for the dairy industry and for other purposes. These nisin variants with additional modification have improved properties and can even expand the inhibition spectrum range of nisin. Nisin, as the most thoroughly studied lantibiotic, and its variants can also guide the modification of other lantibiotics.

## 1. Introduction

Nisin is widely used to treat bovine mastitis, a disease that causes huge economic losses in the dairy industry. Traditional antibiotics have limited utility, and drug-resistant strains lead to milk contamination [1,2]. Nisin is effective against many pathogens causing bovine mastitis, including some with drug resistance [3,4,5]. In addition, nisin has anti-inflammatory actions through activation of the ERK1/2 and p38 mitogen-activated protein kinase (MAPK) signaling pathway and promotes the integrity of the blood–milk barrier [6]. When added to feed, it modulates ruminal microbial ecology, reducing methane production without adversely affecting digestion [7]. However, nisin undergoes proteolytic decomposition in the digestive tract, which is harmless to animal health but reduces its half-life and limits its application as an oral drug. The protein can be used as a food preservative to inhibit pathogens in dairy products and poses no potential health problems for humans [8].

Nisin has also attracted interest as a potential antitumor agent due to its anticancer effect combined with low somatic cytotoxicity. Together with its natural variant, nisin ZP, nisin has been shown to promote apoptosis and inhibit proliferation of head and neck squamous cell carcinoma, colon cancer cells, hepatocellular carcinoma and the chronic myeloid leukemia cancer cell line (K562) [9,10,11] perhaps due to regulation of apoptotic genes [12,13]. The potential immunomodulatory properties of nisin have been discussed in detail in a recent comprehensive review [14].

Nisin is a prototypical type AI lantibiotic encoded by four genes present in clusters *lanABCT* [15]. The structural gene, *lanA*, encodes precursor peptides or prepeptides, and *lanB* and *lanC* encode dehydratase B and cyclase C. The LanB dehydratase catalyzes the dehydration of serine or threonine residues to 2,3-didehydroalanine (Dha) or 2,3-didehydrobutyrine (Dhb) in the LanA structural region, and the LanC cyclase conjugates cysteine residues to these dehydro amino acids to form lanthionine or methyl-lanthionine rings. The fourth gene, *lanT*, encodes an ABC transporter to translocate the modified prepeptide across the cytoplasmic membrane for extracellular modification by NisP. NisP is external to the membrane and removes the leader region to produce mature nisin [16]. *E. coli* has been engineered to synthesize lantibiotics by introduction of the biosynthetic pathway, [17] but production levels are limited by the toxicity of the molecules to the host [18]. This barrier has been overcome by synthesis of a nontoxic prenisin variant that can be transformed into its active form after production [19,20].

**Figure 1:**
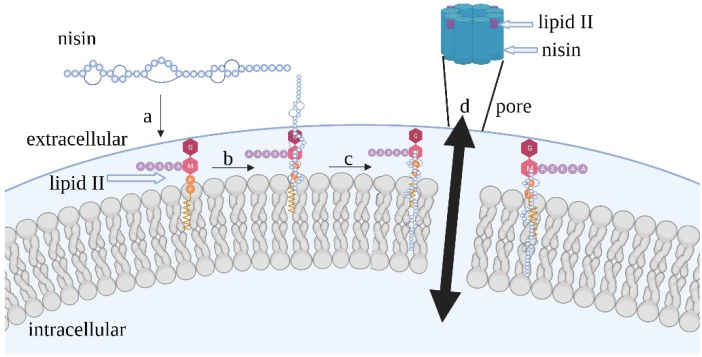
Lipid II-dependent antimicrobial mechanism of nisin. When nisin reaches the cell membrane surface (**a**), rings A and B at the N-terminus of nisin bind to the pyrophosphate group of lipid II (**b**). The C-terminal region inserts into the membrane to form a pore by means of a flexible hinge mechanism (**c**). The pore complex has a stoichiometry of four lipid II molecules to eight nisin molecules (**d**).

Nisin leads to membrane permeabilization via low-affinity membrane permeation plus lipid II-dependent targeted pore formation. The main way for nisin to exert its antimicrobial activity is lipid II-dependent mechanism (Figure 1). Low-affinity membrane permeation is achieved by nisin binding to anionic lipids at mmol concentrations in the target membrane followed by aggregation at the membrane surface to form pores [21]. Lipid II-dependent targeted pore formation is achieved by nisin docking to form a nisin–lipid II complex inserting the C-terminal region by means of a flexible hinge mechanism into the membrane to form a pore. The pore complex has a stoichiometry of four lipid II molecules to eight nisin molecules and inhibits cell wall synthesis, causing leakage of cell contents [22,23]. Nisin recognizes the pyrophosphate moiety of lipid II, unlike vancomycin, which also targets the pentapeptide of lipid II [24]. Vancomycin resistance is generated when the lipid II pentapeptide is modified by the bacterial strain, but pyrophosphate modification would be much more difficult to achieve [25], explaining the rarity of nisin resistance [26]. Moreover, nisin has been found to have antimicrobial activity and anti-biofilm activity against multidrug-resistant *Streptococcus pneumoniae*, methicillin-resistant *Staphylococcus aureus* (MRSA), coagulase-negative *Staphylococci* and vancomycin-resistant *Enterococci* [27,28,29].

However, Gram-negative bacteria lack lipid II, limiting universal antimicrobial activity of nisin. Moreover, nisin stability is pH-sensitive, with optimal bioactivity and solubility occurring at low pH [30], and polymerization at high pH leading to a decline in activity [16,31]. Additional factors, such as instability at neutral pH and fat and emulsifier content limit the antimicrobial effect in dairy products [32,33]. Bioengineering of nisin is aimed at increasing its potency and stability and generating variants with superior properties.

Naturally occurring nisin variants include nisin A and nisin Z, which are common, plus nisin F [34], nisin Q [35], nisin H [36], nisin J [37], nisin U [38] and nisin P [39]. The occurrence of the natural variants indicates which amino acids may be safely substituted without reducing nisin function.

Structural studies of the nisin–lipid II complex [40,41,42] allow predictions to be made for physicochemical properties of variants and their potential interactions with the target molecule by analysis of charge quantity, hydrophobicity and amphiphilicity. Useful tools include Protein Variation Effect Analyzer (PROVEAN) to predict whether amino acid substitutions or indels will influence the biological function of a protein (http://provean.jcvi.org/index.php, accessed on 9 June 2022). In addition, the Antimicrobial Peptide Database (ADP; https://aps.unmc.edu/, accessed on 9 June 2022) has collated 3324 antimicrobial peptides from various organisms, giving structural statistics and 3D structures. The Database of Antimicrobial Activity and Structure of Peptides (DBAASP, https://dbaasp.org/, accessed on 9 June 2022) gives detailed structural information (chemical, 3D) correlated with antimicrobial activity against specific target molecules. This platform uses amino acid sequences to calculate physical and chemical properties and predict antimicrobial activity, and it also allows strain-specific AMPs to be revealed within microbial genomes. Thus, identification of existing antimicrobial motifs and utilization of antimicrobial peptide databases, combined with advanced machine learning algorithms, allow appropriate nisin variants to be designed.

**Figure 2:**
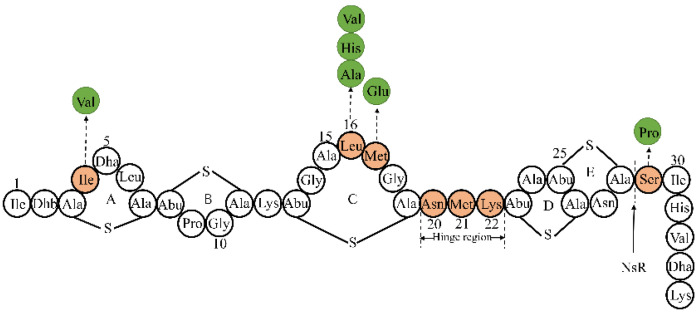
The secondary structure of nisin and some typical valuable variants. Abbreviations: Dha: 2,3-didehydroalanine; Dhb: 2,3-didehydrobutyrine; Abu: 2-aminobutyric acid; NSR: nisin resistance protein. The position indicated by the arrow is the main recognition site of NSR. Light orange-labeled sites are promising mutation sites. There are potential substituted amino acids in the green circles.

Nisin is a small peptide of 34 amino acids and less than 5 kDa, making it a suitable candidate for biological engineering (Figure 2). Modifications have allowed the generation of nisin variants with enhanced antimicrobial activity, solubility, and ability to evade resistance.

We summarized nisin variants with improved anti-pathogen and anti-food spoilage bioactivity and with other enhanced physicochemical properties reported in the previous research. Given the important role of nisin in the dairy industry, transforming nisin to meet the needs of practical applications has become a great trend. Furthermore, these nisin variants with enhanced properties will lead to the discovery of promising new drug candidates.

## 2. N-Terminal Domain Variants of Nisin

The N-terminal binds lipid II with high affinity at nmol concentrations of nisin via interaction between the lipid II pyrophosphate group and main chain amide bonds of nisin, which form a cage-like structure by intermolecular hydrogen bonds [40]. N-terminal amino acid substitutions have been used to elucidate the structural factors controlling molecular recognition of rings A and B [41]. A truncated peptide (1–12) variant (Thr2, Ser5) failed to form the conformation binding lipid II in solution [42]. The structural integrity of rings A and C is essential for antimicrobial activity [43]. Variant (S3T) has an altered N-terminal conformation that affects lipid II binding, increasing the minimum concentration required for pore formation from 1 nM to 50 nM [22]. Most variants obtained by mutation at position 12 of the nisin core peptide had a decreased production level and incomplete post-translational modification, but variant K12A had enhanced antimicrobial activity against representative target strains [44].

Loss of rings D and E impaired antimicrobial activity [43] due to reduced ability to penetrate the cell membrane [45]. Some ring A variants had enhanced antimicrobial activity, such as nisin I4V with more potent antimicrobial and anti-biofilm activity against *Staphylococcus pseudintermedius* [46]. Combination of nisin I4V with conventional antibiotics also increased biological activity against *Staphylococcus aureus* [29]. However, incorporation of large amino acids into ring B had a negative impact on antimicrobial activity. There have also been reports showing no direct correlation between autoinduction and antimicrobial activities of variants [45,47]. Thus, variants with weakened antimicrobial activity but normal inducing activity may be bioengineering targets.

Substitution amino acid physicochemical properties of the first residue of the nisin core peptide [47] have an influence on yield, NisP cleavage efficiency, antimicrobial activity and recognition of nisin immune protein. For example, charged amino acids or those with large hydrophobic side chains at the I1 position reduced the NisP cleavage efficiency [47].

## 3. Hinge Region Variants of Nisin

The flexible hinge region connects the N- and C-terminal regions, and structure–activity studies of nisin and other lantibiotics have identified structural features essential for bioactivity in vivo and in vitro [22,40]. Hinge flexibility is crucial for C-terminal membrane translocation, and the three residues in this region affect stability and solubility. Since variation in this region impacts perforation activity and properties of lantibiotics [22,48], it is a promising target for bioengineering to enhance antimicrobial activity.

Hinge variants evince a wide range of antimicrobial activity. Variants N20K, M21K, N20P, M21V, K22S, and K22T displayed enhanced antimicrobial activity against target strains [49,50,51], but incorporation of aromatic or negatively charged amino acids, serine or threonine in the hinge region negatively impacted biological activity [50].

Saturated hinge mutations result in dramatic changes to nisin structure. Variants generated by this method (SVA, NAK) have been shown to control *Listeria monocytogenes* in complex media (commercially produced chocolate milk), and variants, AAK, NAI, and SLS displayed enhanced antimicrobial activity against various target strains. It can be inferred that small, chiral amino acids in the hinge region are beneficial in enhancing flexibility and thus antimicrobial activity. Variants AAA and SAA, generated by rational design, displayed enhanced activity against target strains [52].

Decreasing hinge region length led to a sharp decrease in antimicrobial activity. Residual activity was probably due to strong cell membrane deformation caused by nisin crowding at the membrane surface through the lipid II-independent antimicrobial mechanism. However, a nisin variant with reduced hinge length (NK) showed enhanced biological activity against *Enterococcus faecalis* at higher temperatures, possibly due to thinning of lipid bilayers as the temperature rises [48,53]. However, the predominant impact of reduced hinge region length is to impair pore formation, leading to increased concentrations being required for antimicrobial activity.

Variants with amino acid insertions (+1, +2 variants) have exhibited some enhanced antimicrobial effects against indicator strains with thicker membranes. Others had stronger antimicrobial activity at low temperatures. However, variants with insertion of three amino acids (+3) displayed dramatically reduced activity under all experimental conditions [48]. The variant _20_NIVMK_24_ retained some antimicrobial activity and was not recognized by nisin-resistant proteins [54], making it an excellent candidate to deal with nisin resistance.

Most hinge region mutations, including insertions and deletions, did not dramatically affect nisin production in host bacteria, but there were some exceptions [48]. For example, production of variants N20D, K22D, K22E and +1 variants _20_NLMK_24_, _20_NVMK_24_, and _20_NIMK_24_ was almost undetectable [49,50,54]. Reduced production may be due to the introduction of negatively charged residues into the hinge region, hindering the formation of ring D [49]. Other workers have shown that the introduction of negatively charged amino acids had no effect on the recognition of NisB and NisC [10].

Decreased hinge region length had a particularly significant impact on posttranslational modification. Dehydration reactions were affected [48], and variant Δ_21_MK_22_ lost its pore-forming activity. Precursor peptide cleavage and formation of lanthionine rings were also affected, indicating impaired recognition by the posttranslational modification enzyme, NisBCP [54].

Hinge region mutations not only affect antimicrobial activity but also alter the antimicrobial spectrum. Variants N20K and M21K displayed antimicrobial activity against Gram-negative *Shigella*, *Pseudomonas* and *Salmonella* [49]. M21F was active against Gram-negative bacteria from the genus Thermus, responsible for higher levels of redness in cheese samples [55]. Nisin variant M21V, alone and in combination with traditional antibiotics, enhanced anti-biofilm activity against *Staphylococcus* [29]. Subsequently, the combination of M21V and plant essential oils was shown to inhibit the Gram-negative bacteria *Cronobacter sakazakii* and *E. coli O157:H7*.

The hinge region plays a key role in biological and pore-forming activity and is a potential target for bioengineering.

## 4. C-Terminal Domain Variants of Nisin

The C-terminal region is involved in pore-forming activity and truncation affects antimicrobial activity to a degree proportional to the length of the truncation [43]. This is also the region of the protein associated with nisin resistance.

Some valuable C-terminal variants include N27K and H31K, which exhibit enhanced solubility at neutral pH, and S29A, S29D and S29E, which had enhanced antimicrobial activity against *Staphylococcus aureus* [16,56]. Nisin resistance protein from *Streptococcus agalactiae* (saNSR) bound to ring E of nisin and cleaved a site between the 28th and 29th amino acid residues of the core peptide [57]. After cleavage by saNSR, activity of the resulting nisin peptides decreased 400–500 fold [58]. However, some C-terminal domain variants evade the resistance mechanisms, and variants S29P and C28P retained considerable antimicrobial activity to inhibit strains expressing nisin immune protein [59,60].

The C-terminal region is also responsible for the initial interaction with the target membrane [21], and incorporation of negatively charged amino acids in this region has been found to adversely impact antimicrobial activity. Variant V32E had an increased minimal inhibitory concentration for *Micrococcus flavus* and *Streptococcus thermophilus* [21]. However, some nisin variants in which negatively charged amino acids were introduced into the C-terminal, such as S29A, S29D and S29E, had enhanced antimicrobial activity against Gram-positive drug-resistant bacteria and some food-borne Gram-negative bacteria.

## 5. Nisin Variants Containing Noncanonical Amino Acids

Standard methods for nisin engineering, such as alanine scanning, site-directed or saturation mutagenesis, have usually incorporated canonical amino acids into nisin variants. Noncanonical amino acids (ncAAs) are non-proteinogenic, except for pyrrolysine (pyl) and selenocysteine, and many different side chain structures and backbone configurations have been generated [61]. The possibilities of proteins containing ncAAs have greatly expanded the potential of nisin engineering.

Both residue-specific and site-specific approaches have been taken to incorporate ncAAs into proteins [62]. A residue-specific approach involves the feeding of amino acid analogues to nutritionally deficient strains to replace canonical residues without gene manipulation [63]. Derivatives of tryptophan, phenylalanine, proline and methionine have all been incorporated into target positions of nisin by selective pressure incorporation (SPI) [63,64,65]. NcAAs can be incorporated into specific sites by means of an orthogonal aminoacyl-tRNA synthetase (aaRS)–tRNA pair. Pyl-tRNA synthetase and its cognate tRNA from the methanogenic archaeon *Methanosarcina mazei* have been used to incorporate Nε-Boc-L-lysine (BocK) and two phenylalanine derivatives into nisin target sites [66,67]. Insertion of methionine derivatives with bio-orthogonal reactivity into nisin enables fluorescence labeling of nisin and coupling of nisin variants [68]. In addition, a variant in which novel macrocyclic topologies replaced the lanthionine ring was derived from reaction between electrophilic ncAAs and cysteine, although no bioactive conformation was formed [65].

Most ncAAs are synthesized by total-chemo or semi-chemo synthesis or extracted from natural substances [69]. The synthesis process of ncAA is complex and expensive, which is not suitable for large-scale production in industry [70]. In vivo synthesis of ncAAs from simple precursors may be a way to overcome the economic barrier and allow the full potential of nisin modified with ncAAs to be realized.

## 6. Nisin-Derived Hybrid Peptides

Hybrid peptides, which combine the sequence of one or more different peptides with nisin, have been synthesized [71] to enhance structural diversity and bypass specific bacterial resistance mechanisms [66,72].

A hybrid incorporating the nisin N-terminal sequence (1–22) responsible for lipid II recognition with vancomycin had a 40-fold enhancement of antimicrobial activity against vancomycin-resistant strains compared with native vancomycin [73]. Similarly, a lantibiotic, TL19, with two lipid II binding motifs, one at the N-terminal from nisin and the other at the C-terminal from haloduracin, has been synthesized. This hybrid peptide had 64-fold higher antimicrobial activity against the difficult-to-treat *Enterococcus faecium* than N-terminal nisin (1–22) and a 2- to 4-fold higher activity against *Enterococcus faecium* than native nisin. The hybrid peptide had no pore-forming activity, demonstrating that the additional lipid II binding sites completely compensated for loss of pore-forming activity [74]. These results look very promising for the development of new lantibiotics.

Structures that are not naturally occurring have also been introduced into peptides to improve antimicrobial properties. Peptoids (N-substituted glycines) have shown antimicrobial activity against microorganisms, fungi and parasites and have higher resistance to proteases than α peptides [72,75]. Nisin-peptoid hybrids containing the nisin lipid II targeting domain have increased in vivo stability and represent a way of increasing the chemical diversity. Significantly, nisin-peptoid hybrids retained the considerable activity of nisin against MRSA [66].

These promising novel structures have the potential to overcome some shortcomings of nisin and reduce drug resistance.

## 7. Production of Nisin Variants

Nisin variants are usually produced by transfection of *E. coli* with the nisin biosynthesis pathway or the expression of a nisin variant gene in a wild-type nisin producing strain from which the nisin gene has been deleted (Table 1). In addition, nisin can be produced by solid-phase peptide synthesis (SPPS), but this is an expensive method unsuitable for large-scale production.

Traditionally, the identification of lantibiotic variant antimicrobial activity has involved a long process of purification and characterization. However, downscaling and parallelization have been achieved. Peptide modules from 12 natural lantibiotics with different antimicrobial mechanisms have been organized according to the structural distribution of nisin and used to generate a combinatorial lantibiotic library containing almost 6000 variants. A high-throughput screening platform may then be used to obtain combinatorial lantibiotics and screen variants with enhanced antimicrobial activity against target pathogens [82].

Host fermentation conditions and expression of post-translationally modifying enzymes may be optimized to increase yield. For example, increasing tRNA^glu^ expression enhanced NisB expression [65], leading to a higher level of nisin variant production. Resistance to nisin may be engineered by heterologously expressing the nisin resistance protein [83].

In vitro activation of nisin precursors prevents nisin poisoning of hosts, and precursor peptides may then be harvested by a series of steps. First, nisin precursor was purified and digested by the endoprotease NisP [59,68]. Second, indicator strains expressing NisP were used to evaluate the antimicrobial activity of nisin variants [63,78]. Third, nisin precursor was purified and digested by trypsin [48,65] but with some inhibition of antimicrobial activity [84]. An excellent, simplified method for removal of the lantibiotic leader sequence involved the incorporation of hydroxy acid into the precursor by pylRS–pylT pair, resulting in an ester linkage connecting the leader peptide, which may be hydrolyzed [19]. More recently, compartmentalization within the bacterial host has been exploited. Biosynthesis and modification of the precursor peptide took place in the cytosol, but lantibiotic release in the periplasmic space. This method is suitable for high-throughput screening of lantibiotic variants and provides an advanced concept for mass production without the additional step of removing the leader peptide [20].

Residue substitutions can also affect nisin variant production levels. The absence of threonine, serine and cysteine or the introduction of negatively charged residues in the core peptide did not affect NisB and NisC recognition, but antimicrobial activity declined sharply, perhaps due to loss of electrostatic interactions with lipid II [10]. Introduction of glutamic acid into the hinge region decreased production dramatically due to disruption of NisB- and NisC-independent steps [49]. Cleavage by NisP depends on the modification of precursor peptides; hence, factors affecting NisB and NisC will also affect NisP cleavage efficiency [85]. Low cleavage rates of the natural variant, nisin H, by NisP were restored by replacing phenylalanine with isoleucine [86], and integrity of the (methyl-)lanthionine rings is a prerequisite for recognition by the cleavage enzyme [85]. Therefore, the yield of nisin variants requires that the impact on residue changes be considered.

**Figure 3:**
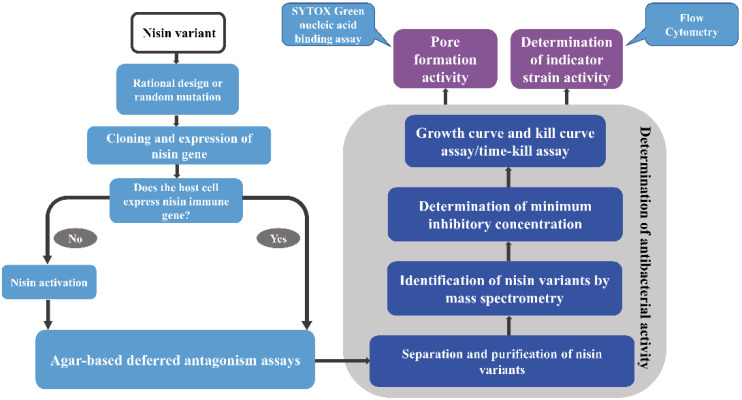
Overview of the approach employed in biosynthesis of nisin variants in different hosts and characterization of nisin variants.

Identification of variant antimicrobial activity requires the following steps (Figure 3). First, appropriate indicator strains for the specific practical application should be selected. Antimicrobial activity should be evaluated using an agar-based deferred antagonism assay. Factors such as temperature, variant solubility and indicator strain types all have an impact on the extent of the inhibition zone and should be taken into account [87] as expansion of the inhibition zone does not necessarily correlate with specific activity. Growth and kill curve assays should be conducted to determine antimicrobial inhibition within the indicator strains [88]. Following laboratory assays, model food trials would assess the antimicrobial agent activity in more complex media of various food types. In addition, the composition or thickness of the bacterial cell wall and plasma membrane content of negatively charged lipids also determine target cell sensitivity to nisin [21].

## 8. Conclusions

The state of knowledge regarding nisin variants, their physicochemical properties and some trends in production was reviewed. Variants enhance our understanding of the structure and organization of nisin and allow progress to be made in overcoming the inherent shortcomings of nisin. Knowledge of structure–function relationships for the constituent domains of nisin facilitates designing protein variants with specific objectives, and improvements in bioengineering technology have made variant synthesis approachable. However, generating a series of nisin variants with enhanced antimicrobial activity still presents a challenge, especially in complex environments.

## Figures and Tables

**Table 1:** Nisin variants produced by bioengineering in different hosts.

Host	Methods	Prenisin	Activation of Prenisin	Nisin Sensitive Indicator	Variants	Variant Features	References
*L. lactis* NZ9800 ^1^	site-directed mutagenesis	No	N/A	*M. flavus*, *S. thermophilus*	ΔN20, ΔM21	inactive in the pore formation assay	[22]
*L. lactis* NZ9800 ^1^	site-directed mutagenesis	No	N/A	*M. flavus* *S. faecalis*, *L. monocytogenes*, *S. cerevisiae*, *G. candidum*.	N20K, M21K	higher solubility, displayed antimicrobial activity against some G− strains	[49]
*L. lactis* NZ9800 ^1^	random mutagenesis, saturation mutagenesis	No	N/A	*L. lactis ssp. cremoris* HP, *E. faecium*, *S. agalactiae*, *S. aureus*, *L. monocytogenes*.	N20P, M21V, K22S	enhanced antimicrobial activity	[50]
*L. lactis* NZ9800 ^1^	random mutagenesis, saturation mutagenesis, site-directed mutagenesis	No	N/A	A series of G+ positive and G− bacteria ^5^	S29A/D/E/Q	enhanced antimicrobial activity against both G+ and G− bacteria	[56]
*L. lactis* NZ9800 ^1^	saturation mutagenesis	No	N/A	*Streptococcus mitis*, *L. lactis* UCC90, *L. lactis* HP, *L. monocytogenes*, *S. agalactiae*	SVA, NAK	enhanced antimicrobial activity against *L. monocytogenes* in complex matrices	[76]
*L. lactis* NZ9800 (*L. lactis* NZ9700ΔnisA)	saturation mutagenesis, site-directed mutagenesis	No	N/A	Six Gram-positive bacteria	K12A	enhanced antimicrobial activity	[44]
*L. lactis* NZ9000	site-directed mutagenesis	Yes	NisP (expressed by indicator strain)	*L. lactis NZ9000* -pNZnisPT	variants of precursor nisin with negatively charged residues ^11^	severe decrease in antimicrobial activity	[10]
*L. lactis* NZ9800 ^1^	saturation mutagenesis	No	N/A	*S. agalactiae*, *L. lactis* HP *M. smegmatis*	AAK, NAI, SLS	enhanced antimicrobial activity	[52]
*L. lactis* NZ9000	site-directed mutagenesis	Yes	trypsin	A series of Gram-positive bacteria ^6^	20NK21, 20NLMK23, 20NVMK23, 20NIMK23 20NIVMK24	enhanced antimicrobial activity against specific strains at certain temperatures	[48]
*L. lactis* NZ9800 ^1^	saturation mutagenesis	No	N/A	*S. pseudintermedius*, *S. intermedius*, *S. aureus*, *L. lactis* HP, *S. uberis*, *B. cereus*	I4V	enhanced antimicrobial activity and anti-biofilm activity against *S. pseudintermedius* and *S. intermedius*	[46]
*E. coli*	ASM ^4^	Yes	trypsin	*L. lactis* NZ9000 NlacZ	L16A, L16H, L16V, M21A, M21D, M21N	increased induction activity and antimicrobial activity	[77]
*L. lactis* NZ9800 ^1^	site-directed mutagenesis	No	N/A	*S. aureus* SA113, *S. pseudintermedius* DSM21284	M21V, I4V	The activity of the nisin derivative and antibiotic combination was higher than that of the nisin and antibiotic combination	[29]
Trp-auxotrophic *Lactococcus lactis* NZ9000	SPI ^2^	Yes	NisP	*L. lactis* MG1363	four different positions of nisin Trp and Trp analogue variants ^12^	Nisin variants containing tryptophan analogues	[64]
*E. coli*	SCS ^3^	Yes	trypsin	*M. flavus*	Ser3TAG	Nisin variant with novel macrocyclic topologies	[65]
Pro-auxotrophic *E. coli* strain	SPI ^2^	Yes	NisP (expressed by indicator strain)	*L. lactis* NZ9000	P9X ^7^	Nisin variant with 6 proline analogues	[63]
*Lactococcus lactis* NZ9000, *E. coli*	SCS ^3^	Yes	NisP (expressed by indicator strain)	*L. lactis* NZ9000	I4BocK ^8^, K12BocK ^8^	enhanced antimicrobial activity	[78]
*E. coli*, C321.ΔprfA-T7RNAPΔrneΔompTΔlon	site-directed mutagenesis	Yes	trypsin	*L. lactis* HP	Ser5m-BrPhe	Nisin variant with Phe analogues	[67]
*L. lactis* NZ9000	site-directed mutagenesis	Yes	NisP	*L. lactis* NZ9000Cm/NisI/NisFEG	_20_NMKIV_24_	decreased recognition of immunity protein	[54]
*L. lactis* NZ9000	saturation mutagenesis	Yes	NisP	*L. lactis* NZ9000-Cm/NisI/NisFEG/SaNSR /SaNsrFP	I1X ^9^	I1X variants influenced antimicrobial activity and the efficiency of the immunity and resistance proteins.	[47]
*L. lactis* NZ9800 ^1^	saturation mutagenesis	No	N/A	*L. lactis subsp. diacetylactis* DRC3 (expressing the nisin resistance protein (NSR))	S29P	The variant exhibited a 20-fold increase in specific activity against a strain expressing the nisin resistance protein.	[60]
*L. lactis* NZ9000	site-directed mutagenesis	Yes	NisP	*L. lactis* NZ9000 pNZ-SV-Erm/SaNSR/SaNSRS236A	C28P	3 times more efficient against SaNSR-expressing *L. lactis* cells	[59]
*L. lactis* NZ9800 ^1^	site-directed mutagenesis	No	N/A	*Lb. plantarum* UCC16, *Lb. brevis* SA-C12, *L. lactis ssp. cremoris* HP	P9T, P9S	The variants retain induction capacity, while most of the antimicrobial activity is abolished.	[79]
Met-auxotrophic *Lactococcus lactis* NZ9000	SPI ^2^	Yes	NisP	*L. lactis* and six Gram-positive pathogenic strains ^10^	M21V-M17Aha + M21VM17Hpg	The variant is the most active dimeric nisin construct	[68]
*L. lactis* NZ9800 ^1^	site-directed mutagenesis	No	N/A	*L. innocua FH1836lux*	M17Q + N20P, M17Q + S29E	The combinations of nisin derivative exhibited enhanced anti-listerial activity when used together compared to when used alone	[80]
*L. lactis* NZ9000	site-saturation mutagenesis	Yes	Not mentioned	*S. aureus* RF122, *S. aureus* NCDO1499, *S. agalactiae* ATCC13813, *L. lactis* HP, *L. lactis* MG1363	M17Q, T2L, HTK	improved specific activity against some *Staphylococci* but unchanged or reduced activity against dairy *Lactococci*	[81]
*L. lactis* NZ9800 ^1^	site-directed mutagenesis	No	N/A	*T. thermophilus* HB27, *T. scotoductus* Se-1	M17Q, M21F	enhanced specific activity against Thermus strains	[55]

^1^*L. lactis* NZ9800: *L. lactis* NZ9700∆nisA. ^2^ SPI = selection pressure incorporation. ^3^ SCS = stop codon suppression. ^4^ ASM = alanine scanning mutagenesis. ^5^ Targets included the antibiotic resistant *S. aureus* strains ST 528 (MRSA), ST 530 (MRSA), hVISA 32679, as well as *S. aureus* RF122, *Streptococcus mitis*, *L. lactis* HP and MG1363, *Bacillus cereus* DPC 6088/6089, *Enterococcus durans* and *L. monocytogenes* strains 10403S and LO28. ^6^ *Enterococcus faecalis* VE14089, *Listeria monocytogenes*, *Bacillus cereus* 4147, *Bacillus cereus* 4153, *Lactococcus lactis* MG1363, *Bacillus cereus* (L’29) 16, *Micrococcus luteus*, *Streptococcus pneumoniae* R6, *Bacillus sporothermodurans* lC4, *S. aureus*. ^7^ X represents six proline analogues: (4R)-fluoroproline, (4R)-hydroxyproline, (4R)-methanoproline, (4S)-fluoroproline, (4S)-hydroxyproline, (4S)-methanoproline. ^8^ BocK = Nε-Boc-L-lysine charged amino acids. ^9^ X includes four classes depending on the amino acid property: (1) aliphatic amino acids (L, A, V, G); (2) aromatic amino acids (W, F, Y); (3) C, T, S; (4) charged amino acids (K, R, H, Q, E, N, D). ^10^ The tested strains included two *Staphylococci*, two *Enterococci*, *Bacillus cereus* and *Listeria monocytogenes*. ^11^ NisA-H6 T2D P9D with two negatively charged residues, NisA-H6 T2D P9D K12D N20E with four negatively charged residues, and NisA-H6 T2D P9D K12D N20E H27D K34E with six negatively charged residues. ^12^ Tryptophan and tryptophan analogue variants at four positions of nisin: I1W/5FW/5HW/5MeW, I4W/5FW/5HW/5MeW, M17W/5FW/5HW/5MeW, V32W/5FW/5HW/5MeW.

## Data Availability

Not applicable.
